# Detection of the Quarantine Species *Thrips palmi* by Loop-Mediated Isothermal Amplification

**DOI:** 10.1371/journal.pone.0122033

**Published:** 2015-03-20

**Authors:** Arnika Przybylska, Żaneta Fiedler, Halina Kucharczyk, Aleksandra Obrępalska-Stęplowska

**Affiliations:** 1 Interdepartmental Laboratory of Molecular Biology, Institute of Plant Protection—National Research Institute, Poznań, Poland; 2 Department of Biological Control, Institute of Plant Protection—National Research Institute, Poznań, Poland; 3 Department of Zoology, Maria Curie-Skłodowska University, Lublin, Poland; University of Brighton, UNITED KINGDOM

## Abstract

*Thrips palmi* (from the order Thysanoptera) is a serious insect pest of various crops, including vegetables, fruits and ornamental plants, causing significant economic losses. Its presence constitutes a double threat; not only does *T*. *palmi* feed on the plants, it is also a vector for several plant viruses. *T*. *palmi* originated in Asia, but has spread to North and Central America, Africa, Oceania and the Caribbean in recent decades. This species has been sporadically noted in Europe and is under quarantine regulation in the European Union. For non-specialists its larval stages are indistinguishable morphologically from another widespread and serious insect pest *Frankliniella occidentalis* (a non-quarantine species in the European Union) as well as other frequently occurring thrips. In this study, we have developed a loop-mediated isothermal amplification protocol to amplify rDNA regions of *T*. *palmi*. The results were consistent whether isolated DNA or crushed insects were used as template, indicating that the DNA isolation step could be omitted. The described method is species-specific and sensitive and provides a rapid diagnostic tool to detect *T*. *palmi* in the field.

## Introduction

Thrips, members of the order Thysanoptera, are tiny, slender insects with fringed wings. Thrips are common plant pests of various crops. They feed by puncturing the epidermal (outer) layer of host tissue and sucking out the cell contents, which results in stippling, discolored flecking, or silvering of the leaf surface. Thrips feeding is usually accompanied by black varnish-like fecal droplets. Thrips cause discoloration of leaf, flower, and fruit surfaces and distortion of plant parts. They can vector plant pathogens, including viruses of quarantine importance. The plesiomorphic character state which distinguishes Thysanoptera from other insect orders is an asymmetric mouth cone. About 6000 species of thrips have been identified throughout the world, around 600 of these have been reported in Europe, from which about 220 occur in Poland [1]. Most of these thrips species are plant-feeding insects and a group of these being crop pests. The most harmful species are: *Thrips tabaci* Lindeman, *Frankliniella occidentalis* (Pergande), and *Thrips palmi* Karny [2]. The latter is under quarantine regulation in the European Union [3]. The spread of these species is limited by the application of biological and chemical control methods. It is noteworthy that chemical control is relatively efficient in the case of *T*. *tabaci* because all of this insects developmental stages usually occur on the green parts of plants [4]. The situation is different in the case of *F*. *occidentalis* and *T*. *palmi*, where the development of pupal stages usually takes place in the soil and this stage constitutes about 40% of the length of a full life cycle [5, 6]. During this time, these insects are virtually inaccessible to the majority of chemicals and furthermore biological control is only partly effective for these pests [7, 8]. Importantly, another problem in combating these serious pests is the difficulty in distinguishing non-quarantine *F*. *occidentalis* and the more harmful European Union quarantined insect, *T*. *palmi*, in their larval stages. Determination of the thrips species infecting a crop is therefore an important element in plant protection strategies. *T*. *palmi* is a polyphagous pest that particularly effects Cucurbitaceae and Solanaceae plants. It appears to have originated from South-Eastern Asia and to have spread from there during the 20th century [9]. It is now present throughout Asia, North and South America, Central America and the Caribbean, Africa and Oceania. It is not present in Europe but there have been several outbreaks in the Netherlands which were subsequently eradicated [10]. There are six or seven stages in the life cycle of thrips: the egg, three (Terebrantia) or four (Tubulifera) pre-imaginal stages and the adult stage.

Distinction of the different species of thrips at the morphological level requires entomological expertise. However, more and more insects, including those for which diagnostics is difficult, can be detected using molecular biology tools [11–14]. There are also methods described for the molecular identification of *T*. *palmi*. These include standard and real-time PCR detection protocols using primers hybridizing to the mitochondrial cytochrome oxidase subunit (*mtCOI*) gene [15], SCAR marker-based real-time PCR detection using a Taqman probe [16] and PCR-RFLP detection using primers amplifying the ITS2 region of rDNA [17]. To ensure faster diagnostics, multiplex PCR methods are being developed which enable the identification of various samples (*e*.*g*. various species) in one reaction [18]. However, to allow fast detection without extensive laboratory equipment, more and more organisms and non-organisms are being detected using loop-mediated isothermal amplification (LAMP).

The LAMP method relies on auto-cycling strand displacement DNA synthesis that is performed by a DNA polymerase with high strand displacement activity and a set of specially designed primers. LAMP amplifies DNA with high efficiency under isothermal conditions [19] and reaction products can be visualized using DNA intercalating dyes (*e*.*g*. SYBR Green or EvaGreen) which can be added after the reaction and fluorescence may be observed under a UV lamp. Therefore, the method is simple and applicable under non-laboratory conditions because only a water bath (or heated block) and UV lamp are required.

In this study, we have developed a LAMP-based diagnostic method for the detection of the quarantine *T*. *palmi* insect species using primers amplifying its rDNA sequence. The method is sensitive and species-specific and will provide an effective diagnostic tool for use in the field.

## Materials and Methods

### Insect samples

Thrips populations of 11 *Thrips* species and four *Frankliniella* species, listed in the table below (Table 1), were used in this study. The species originated from Poland were reared under laboratory conditions on their host plants. Specimens of each species, both adults and larvae, were mounted in Berlese fluid on microscopic slides and identified according to Strassen [20] and Vierbergen et al. [21]. The specimens (both adults and larvae) of each species to be used for molecular analysis were stored separately in labeled tubes containing absolute ethyl alcohol.

**Table 1:** Thrips populations used in this study.

Species	Origin	Host plant	GenBank accession number
*Thrips palmi*	Japan	*Phaseolus vulgaris*	KM877305
*Thrips palmi*	Taiwan	*Cucumis sativus*	KM877306
*Thrips palmi*	Taiwan	*Citrulus lanatus*	
*Thrips tabaci*	Poland	*Cucumis sativus*	KM877307
*Thrips tabaci*	Poland	*Inula salicifolia*	KM877308
*Thrips major*	Poland	*Sambucus nigra*	KM877309
*Thrips menyanthidis*	Poland	*Menyanthes trifoliata*	
*Thrips nigropilosus*	Poland	*Ocimum basilicum*	-
*Thrips origani*	Poland	*Origanum vulgare*	-
*Thrips physapus*	Poland	*Centaurea jacea*	-
*Thrips roepkei*	Poland	*Solanum dulcamara*	KM877310
*Thrips simplex*	Poland	*Gladiolus* sp.	KM877312
*Thrips sambuci*	Poland	*Sambucus nigra*	KM877311
*Thrips trehernei*	Poland	*Tragopogon pratensis*	KM877313
*Frankliniella occidentalis*	Poland	*Cucumis sativum*	KM886242
*Frankliniella intonsa*	Poland	*Gentiana pneumonanthe*	KM886243
*Frankliniella pallida*	Poland	*Sedum acre*	KM886244
*Frankliniella tenuicornis*	Poland	*Zea mays*	KM886245

*Thrips palmi* specimens originated from various locations and various host plants (Table 1). Before molecular analysis, specimens were morphologically analyzed and classified into species. The tests (sequencings) described below also confirmed genetic differences between them (several substitutions in the analyzed rDNA region). The reference materials were deposited in the collection of the Department of Zoology, Maria Curie-Skłodowska University in Lublin, Poland. The animal work carried out in this study was conducted according to the relevant national and international guidelines.

### Total genomic DNA extraction

DNA extraction was performed using a NucleoSpin Tissue kit (Macherey-Nagel, Düren, Germany). For each extraction, individual insects were used (both the larvae and adult stages), with a final elution volume of 50 μl and a DNA concentration of about 1 ng/μl.

### Sequence analysis and primer design

The sequences of the 18S-ITS1–5.8S-ITS2–28S rDNA regions from many thrips species (*T*. *palmi*, *T*. *tabaci*, *T*. *coloratus*, *T*. *havaniiensis*, *T*. *setosus*, *Frankliniella occidentalis*, *F*. *intonsa*, *F*. *schultzei*, *Scirtothrips dorsalis*, *Echinothrips americanus*) were collected from the GenBank database (S1 Table), then aligned and analyzed using the BioEdit software [14]. A multiple sequence alignment was generated (S1 Fig.) and regions sharing the highest levels of identity among all species were used in the design of universal forward and reverse primers. Primers were checked using the OligoAnalyzer 3.1 tool. The sequences of all primers used in the study are listed in the Table 2.

**Table 2:** Primers used in the PCR reactions and LAMP assay.

Primer	Sequence	Annealing region
**PCR assay**		
thrUNIFw	CGTACAAGGTTTCCGTAGG	18S rDNA
thrUNIRw	GTRRTCTCDCCTGAACWG	28S rDNA
**LAMP assay**		
F3	ATTCCATGCCATCCTTCG	ITS2 rDNA
B3	TAGTCTCACCTGAACAGAGG	28S rDNA
FIP(F1c+F2)	CACTCTGTCCTGCCGCTAAGCGCTTCTGAAGTGGATCTC	ITS2 rDNA
BIP(B1c+B2)	TCGATGTTGTATTGGCATCCGTAAGAGATCTGGCTAGGATCTT	ITS2 rDNA
LoopF	GTAAATCGGAGCGAGGAGG	ITS2 rDNA
LoopB	CGTTCTCTGTAGTCGGAGC	ITS2 rDNA

### PCR reactions using universal primers

Universal primers thrUNIFw/thrUNIRw were tested in PCR reactions with all of the analyzed populations. All reactions were performed in 10-μl final volumes containing 1 μl of DNA, 200 μM of dNTPs, 1 μM of each primer, 1× Allegro*Taq* polymerase buffer, and 0.2 U of Allegro*Taq* polymerase (Novazym, Poznan, Poland) as well as sterile distilled water. The thermal profile was as follows: an initial denaturation step at 95°C for 3 min, then 35 cycles of denaturation (30 s at 95°C), annealing (30 s at 53°C), and extension (90 s at 72°C), and a final extension step for 5 min at 72°C. PCR products were then electrophoresed on 1% agarose gels and visualized with Midori Green stain (Nippon Genetics Europe, Dueren, Germany).

### DNA cloning and sequencing

After electrophoretic separation, the obtained PCR DNA fragments were isolated from gels using the Wizard SV Gel and PCR Clean-Up System (Promega, Madison, WI, USA). The purified fragments were then cloned into pGEM T-Easy vector (Promega) with an overnight ligation step at 4°C and transformed into a chemically competent strain of *E*. *coli* bacteria MAX Efficiency DH10B (Invitrogen, Carlsbad, CA, USA). Clones containing inserts were identified using an α-complementation test and confirmed by PCR using the universal primers. Plasmids from positively selected clones were isolated after overnight incubation in LB medium using NucleoSpin Plasmid (Macherey-Nagel) and automatically sequenced.

### Sequence and phylogenetic analyses

Multiple sequence alignments of sequences from the positive clones were generated using the BioEdit software [22] with default options, and were then edited manually using GeneDoc [23]. To estimate the genetic distance between species, thrips sequences from this study were aligned with rDNA sequences derived from other thrips species collected from the NCBI databases. Phylogenetic analyses were performed using the Neighbor-Joining algorithm in the MEGA4 software [24] (S2 Fig.).

### LAMP primer design

**Fig 1:**
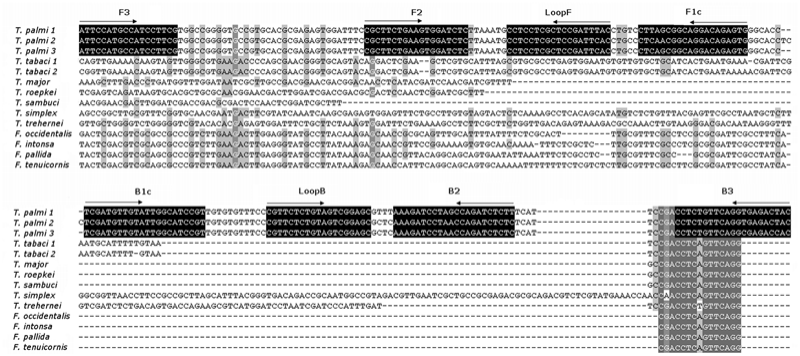
Multiple sequence alignment of the rDNA region of analyzed thrips species. The loop-mediated isothermal amplification (LAMP) primer sequences are indicated (black background). The alignment was generated using the GeneDoc software [23].

LAMP primers suitable for *T*. *palmi* detection were designed using the LAMP designer software PrimerExplorer (http://primerexplorer.jp). Primer sequences were checked using BLAST (Basic Local Alignment Search Tool) against all species belonging to the subfamily Thripinae to exclude cross reactions with other closely related species and ensure the specific detection of *T*. *palmi*. Then, the primers sequences were compared with sequences of *T*. *palmi* populations deposited in the GenBank database, again to ensure specificity to this species (Fig. 1, S1 Table, S3 Fig.).

### LAMP assay

LAMP reactions were performed with 1 μl of thrips species genomic DNA (~ 1 ng/μl), Isothermal Master Mix (OptiGene, Horsham, UK), 0.8 μM of primers FIP and BIP, 0.4 μM of Loop primers and 0.2 μM of primers F3 and B3 (Table 2), and sterile distilled water to a final volume of 25 μl. As a negative control, DNA from thrips species other than *T*. *palmi* (Table 1) were used. A no template control was also included to exclude the possibility of reagent contamination. Reactions were carried out at 63°C for 30 min. The LAMP reaction was performed in two ways: (1) using a real-time PCR platform, Rotor-Gene 6000 (Corbett Life Science, Sydney, Australia), with a fluorescent dye-containing master mix, and (2) in a heating block followed by the addition of 0.5 μl of the final reaction to 20 μl of 1× EvaGreen stain solution (Biotium, Hayward, CA, USA). Fluorescence was then observed under a UV lamp. Products were additionally electrophoresed in a 1% agarose gel then visualized with Midori Green stain (Nippon Genetics Europe). Each population was tested in triplicate.

### LAMP sensitivity test

The sensitivity of the LAMP reaction was tested by performing reactions with nine 10-fold dilutions of *T*. *palmi* genomic DNA extracted from one specimen (starting from 1 μl from 50 μl extract, corresponding to 2 x 10^-2^). Assays were separately performed for the larvae and adult stages.

To further confirm the sensitivity and simplicity of the method, thrips samples without the DNA extraction step were tested. Two populations of *T*. *palmi* were chosen for this test, as well as one *T*. *tabaci* population as a negative control. From each population five individuals were tested separately (from adult and larvae stages). Each specimen was crushed in 50 μl of sterile distilled water, incubated in a water bath at 95°C for 5 min, chilled on ice, and then 1 μl was used as template for the LAMP reaction.

## Results

### PCR reactions with universal primers

We first analyzed rDNA sequences obtained from *T*. *palmi* populations in our study, along with sequences from other thrips species available in the GenBank database. We identified conserved regions in the rDNA of *T*. *palmi* that could be used to differentiate this species from other thrips species and designed universal primers. From PCR reactions, amplification products for the following species were obtained with products sizes as specified: *T*. *palmi—*1500 bp, *T*. *tabaci*—1550 bp, *T*. *major—*1850 bp, *T*. *roepkei—*1350 bp, *T*. *sambuci*—1320 bp, *T*. *simplex*—1660 bp, *T*. *trehernei*—1280 bp, *F*. *occidentalis—*1350 bp, *F*. *intonsa*—1270 bp, *F*. *pallida*—1250 bp and *F*. *tenuicornis*—1290 bp. The amplified products were extracted from gels, cloned into pGEM vectors and transformed to *E*. *coli* cells. The inserts in the recombinant clones were subsequently sequenced. The sequences were deposited in the GenBank database under the accession numbers shown in Table 1.

### LAMP primer target analysis

Next, LAMP primers were designed for *T*. *palmi* detection (Table 2). BLAST analysis of the primers was performed to exclude potential cross-reaction with non-target species. The LAMP primers were shown to hybridize specifically to *T*. *palmi* sequences deposited in the GenBank database, with the exception of primer B3, which was common for most of the species (S3 Fig.).

### LAMP assay

**Fig 2:**
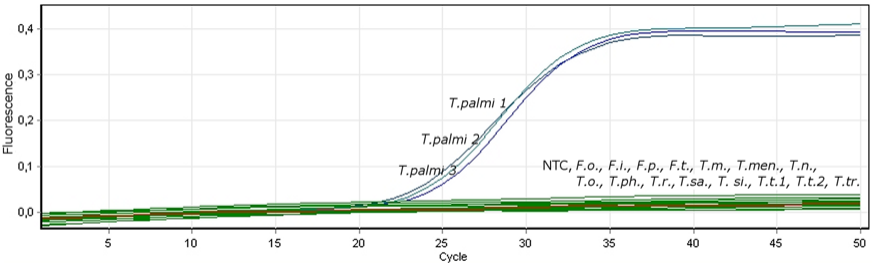
Results of LAMP reaction using real-time monitoring. F.o.—*Frankliniella occidentalis*, F.i.—*F*. *intonsa*, F.p.—*F*. *pallida*, F.t.—*F*. *tenuicornis*, T.m.—*Thrips major*, T.men.—*T*. *menyanthidis*, T.n.—*T*. *nigropilosus*, T.o.—*T*. *origani*, T.ph.—*T*. *physapus*, T.r.—*T*. *roepkei*, T.si.—*T*. *simplex*, T.sm.—*T*. *sambuci*, T.t.—*T*. *tabaci*, T.tr.—*T*. *trehernei*, NTC—no template control.

**Fig 3:**
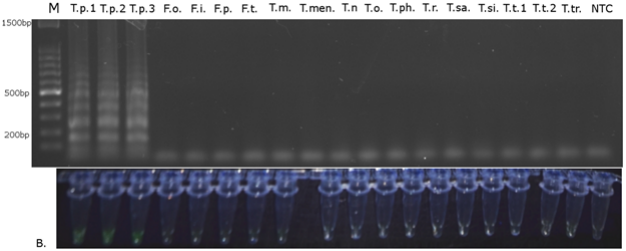
Visualization of LAMP reaction results. A) After electrophoresis in a 1% agarose gel; B) using EvaGreen stain (Biotium). T.p.—*T*. *palmi*, F.o.—*F*. *occidentalis*, F.i.—*F*. *intonsa*, F.p.—*F*. *pallida*, F.t.—*F*. *tenuicornis*, T.m.—*T*. *major*, T.men.—*T*. *menyanthidis*, T.n.—*T*. *nigropilosus*, T.o.—*T*. *origani*, T.ph.—*T*. *physapus*, T.r.—*T*. *roepkei*, T.si.—*T*. *simplex*, T.sm.—*T*. *sambuci*, T.t.—*T*. *tabaci*, T.tr.—*T*. *trehernei*, NTC—no template control.

The LAMP reaction was first performed in a real-time PCR machine using the conditions described above. Only *T*. *palmi* populations gave products in real-time monitoring (Fig. 2). The LAMP assay was also performed in a heating block and 0.5 μl of the final reaction mixture was added to fluorescent dye solution and visualized under a UV lamp. The remaining reaction mixture was subjected to agarose gel electrophoresis. Using this approach, the fluorescence and products on the gel were only observed in samples containing *T*. *palmi* DNA (Fig. 3) in all cases. Using both methods, no products were observed in samples with DNA from other thrips or in the no template control.

### LAMP sensitivity and reproducibility

**Fig 4:**
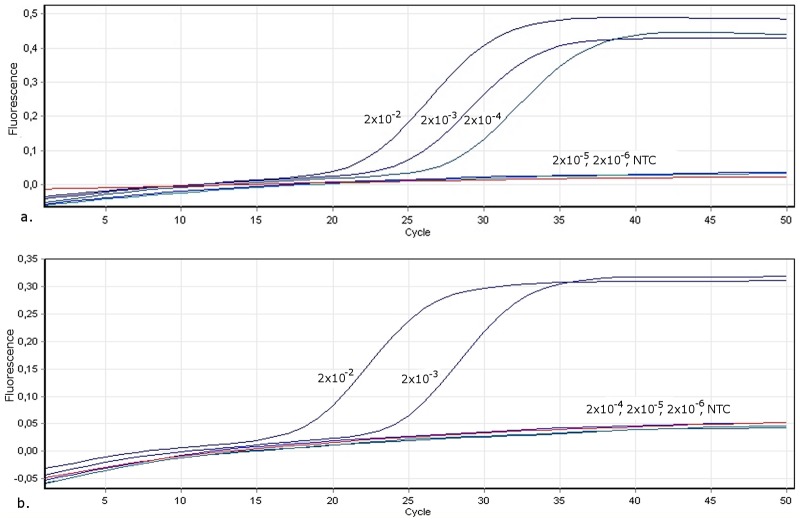
Results of LAMP reactions using real-time monitoring with different dilutions of DNA template. a) DNA extracted from one larva; b) DNA extracted from one adult specimen. NTC—no template control.

**Fig 5:**
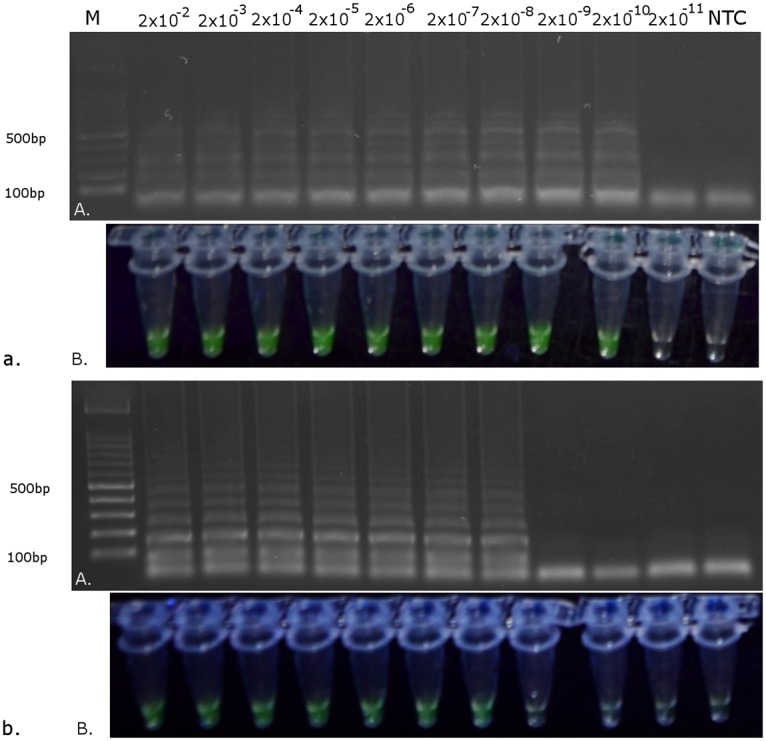
Visualization of LAMP reaction results with different dilutions of DNA template. A) After electrophoresis in a 1% agarose gel; B) using EvaGreen stain (Biotium). a) DNA extracted from one larva; b) DNA extracted from one adult specimen. NTC—no template control.

**Fig 6:**
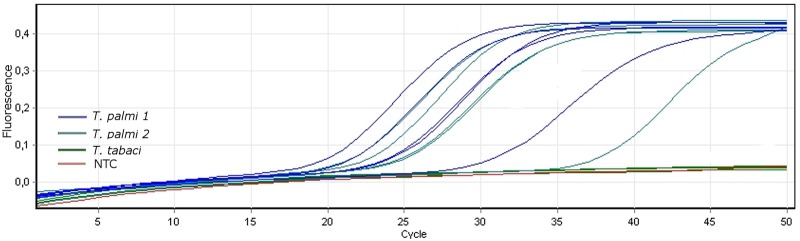
Results of LAMP reactions using real-time monitoring without DNA extraction. T.p.—*T*. *palmi*, T.t.—*T*. *tabaci*. NTC—no template control.

**Fig 7:**
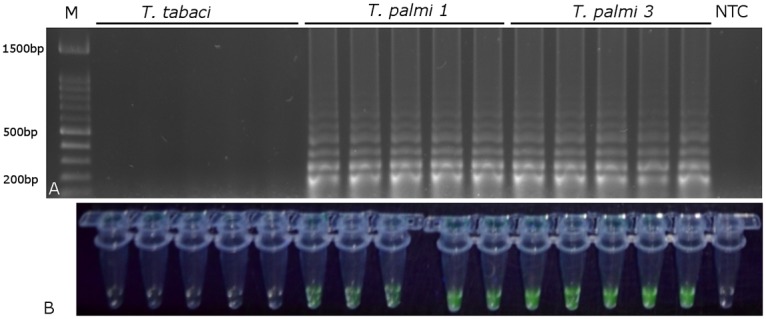
Visualization of LAMP reaction results without DNA extraction. A) After electrophoresis in a 1% agarose gel; B) using EvaGreen stain (Biotium). T.p.—*T*. *palmi*, T.t.—*T*. *tabaci*. NTC—no template control.

The sensitivity test showed that the reaction performed using the real-time PCR platform could detect 2 x 10^-4^ of adult specimen (Fig. 4a) and 2 x 10^-3^ of larvae specimen (Fig. 4b), whereas the reaction performed in the heating block and visualized using EvaGreen stain (Biotium) could detect 2 x 10^-10^ of adult specimen (Fig. 5a) and 2 x 10^-8^ of larvae specimen (Fig. 5b). In reactions performed without the DNA extraction step, fluorescence and gel products were observed for both *T*. *palmi* populations in all replicates (Figs. 6, 7). No products were observed when *T*. *tabaci* was used as template or in the no template control.

In conclusion, the method showed 100% reproducibility with all of the positive samples being detected under all of the conditions analyzed (on the real-time PCR platform, in the heating block followed by electrophoresis, in the heating block following by the observation of green fluorescence after EvaGreen supplementation, and for the crushed *T*. *palmi* sample without the DNA isolation step). The method also showed high sensitivity (up to 10^-10^ of the specimen) and high specificity with no cross-reaction being detected.

## Discussion

The genera *Thrips* and *Frankliniella* are associated mainly with dicotyledonous plants. Among them some species are recognized as pests which can feed both on leaves and flowers, damaging their host plants by puncturing and sucking their cells. Some of them, like *T*. *palmi*, *T*. *tabaci* and *F*. *occidentalis*, can be vectors for plant tospoviruses. *T*. *palmi* is known to transmit a few different plant viruses, including water melon silver mottle virus (WSMoV) [25, 26] and tomato spotted wilt virus (TSWV) [27]. Melon yellow spot virus (MYSV) has also been reported to be transmitted by *T*. *palmi* on melon and cucumber [28].

Distinguishing the different species of thrips morphologically is difficult. Members of the *Thrips* genus have only two pairs of ocellar setae. The antennae are 7- or 8-segmented and segments III and IV have forked sense cones. The pronotum has two pairs of major postero-angular setae. The tarsi are 2-segmented. The abdominal tergites V–VIII have paired ctenidia laterally and the sternites and pleurotergites are set with or without accessory setae [29]. Bhatti [30] and Palmer [31] published reliable guidelines for the morphological identification of *T*. *palmi* occurring in the Asian tropics. Mound & Kibby [32] provided guidelines for the morphological identification of 14 species of economic importance. Nakahara [33] provided guidelines for the identification of species from the Americas. Detailed descriptions of *T*. *palmi* are given by Bournier [34], Sakimura *et al*. [35] and Strassen [36]. Problems, however, are often encountered when preimaginal stages are to be distinguished. Only the second instar larvae of some species of the *Thrips* and *Frankliniella* genera may be recognizable [21]. Then, the most rapid and reliable solution is to carry out molecular investigations. This is particularly important for those plant pests that might negatively impact agroeconomics. The rapid detection of a particular pest might enable effective crop protection to be instigated. Furthermore, special consideration needs to be taken for those pests that owing to their quarantine status need to be rapidly eliminated or contained, as is the case for *T*. *palmi*.

For the molecular detection of *T*. *palmi*, several methods have been developed to date. These methods have been based on PCR using Taqman probe reactions for the amplification of specific marker sequences, such as mitochondrial cytochrome oxidase, the ITS2 fragment of rDNA and SCAR marker. Although identification of *mtCOI* or *II* sequences has proved useful for the detection of other insect species (*e*.*g*. [37, 38]), recently several variants of *mtCOI* were reported in *T*. *palmi* [39] that might result in ambiguity of detection with this marker. Therefore, in this study, the rDNA fragment was chosen as the target for LAMP primer hybridization (Table 2). To exclude cross-hybridization with non-target species, BLAST searches were carried out. These searches confirmed that the designed primers should amplify only *T*. *palmi* populations. Additionally, several other thrips were used in our experimental analyses, especially those frequently found in fields or glasshouses in Europe, *e*.*g*. polyphagous species feeding mainly on dicots: *F*. *occidentalis*, *F*. *intonsa*, *T*. *nigropilosus* and *T*. *tabaci*, as well as those frequently and numerously present on monocots: *T*. *simplex* and *F*. *tenuicornis* (the former on *Gladiolus* sp., the latter on cereals). Also their genetic distance was taken into account and after phylogenetic analysis of sequences available in the GenBank database for rDNA from *Thrips* and *Frankliniella* genera, the most closely related species and the least closely related species were used as controls in the LAMP reaction. Each reaction was performed in triplicate for reactions with DNA template and five replicates were performed for reactions using crushed individuals as template with no DNA extraction step. The reproducibility of the methods was 100%, with all samples containing *T*. *palmi* or its DNA giving positive reaction results. No amplification products were obtained for the negative controls. These findings confirmed that this LAMP method is effective and highly specific for the early detection of *T*. *palmi*. Moreover, it can be used to identify all stages of the life cycle, including the preimaginal stage, which otherwise can only be differentiated from other frequent but non-quarantine species by experts familiar with thrips biology and morphology.

The sensitivity of LAMP is much higher than that of other similar molecular detection methods. For example, using one of the real-time PCR protocols mentioned above [16], it was possible to detect 10^-3^ thrips specimens but the detection threshold using our LAMP assay was much lower, allowing for the detection of 2 x 10^-10^ adults and 2 x10^-8^ larvae (Fig. 5). Although our study only included some of the available thrips species as negative controls, BLAST analysis of all rDNA sequences of the Thripinae subfamily revealed that the primers hybridized specifically to the *T*. *palmi* population sequences present in the GenBank database. By aligning the *T*. *palmi* sequences available in public databases we were able to identify a conserved region of the rDNA that enabled us to design *T*. *palmi-*specific LAMP primers that should be effective in detecting populations of this species around the world (S3 Fig.).

A significant advantage of our proposed method is its potential to be applied both in the laboratory and in the field. Our findings indicate that even the DNA isolation step can be omitted, negating the need for a centrifuge. Consistent results are obtained using crushed insects as template.

Reaction progress can be observed using real-time PCR by the observation of a logarithmic increase in fluorescence over the amplification curve for positive samples (Fig. 2). Additionally, the reaction result can be observed by agarose gel electrophoresis, with amplification products only being present for positive samples (Fig. 3A). The most straightforward way to analyze the LAMP reaction is visualization under a UV lamp, where fluorescence appears only in positive samples containing *T*. *palmi* DNA (Fig. 3B).

## Conclusion

Thrips species both of the *Thrips* and *Frankliniella* genera share some morphological characteristics that make distinguishing them a challenge. Moreover, polyphagous species often feed and breed on the same hosts, which can lead to their misidentification by non-specialists. The experimental approach reported in this study for the detection of the quarantine insect pest *T*. *palmi* might therefore serve a wide-range of diagnosticians working in phytosanitary services.

## Supporting Information

S1 Fig Multiple sequence alignment of rDNA region of thrips species collected from the GenBank database. PCR primer sequences are indicated (black background). The alignment was generated using the GeneDoc software [23].
(TIF)

S2 Fig Phylogenetic analysis of the 5,8S-ITS2–28S rDNA region of selected thrips species using the neighbor-joining algorithm. MEGA4 software [24] was used. Genetic distance is indicated.
(TIF)

S3 Fig Multiple sequence alignment of the rDNA region of *Thrips palmi* sequences in the GenBank database. Loop-mediated isothermal amplification (LAMP) primer sequences are indicated (black background). The alignment was generated using the GeneDoc software [23].
(TIF)

S1 Table Sequences from the GenBank database used for designing PCR and LAMP primers. (DOCX)
