# Supplementary figures and images for: Detection of the Quarantine Species Thrips palmi by Loop-Mediated Isothermal Amplification
Source: PLoS One. 2015 Mar 20;10(3):e0122033. doi: 10.1371/journal.pone.0122033 (PMC4368663; doi:10.1371/journal.pone.0122033)

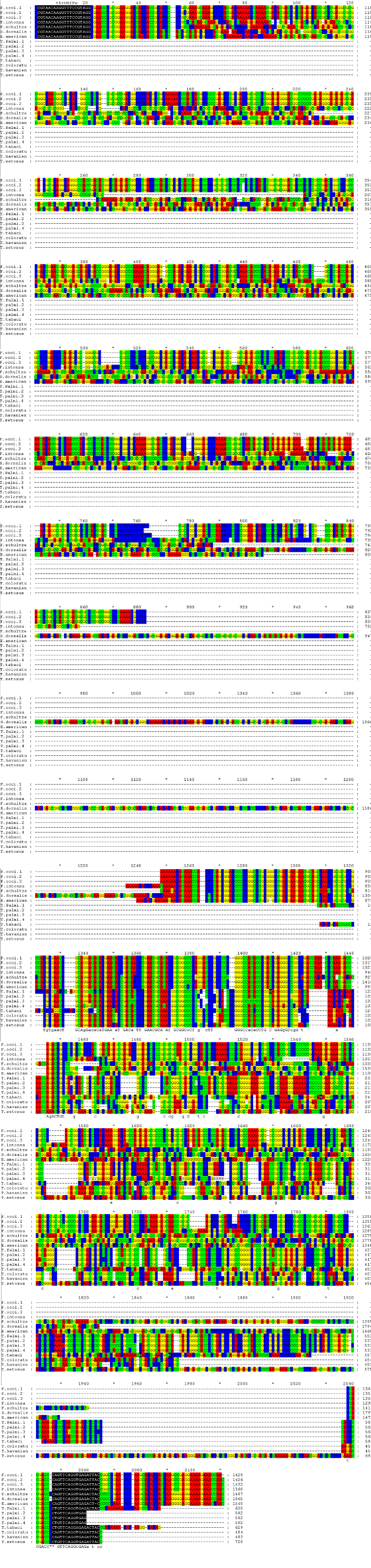

Supplement: S1 Fig — PCR primer sequences are indicated (black background). The alignment was generated using the GeneDoc software [23]. (TIF) [file pone.0122033.s001.tif]

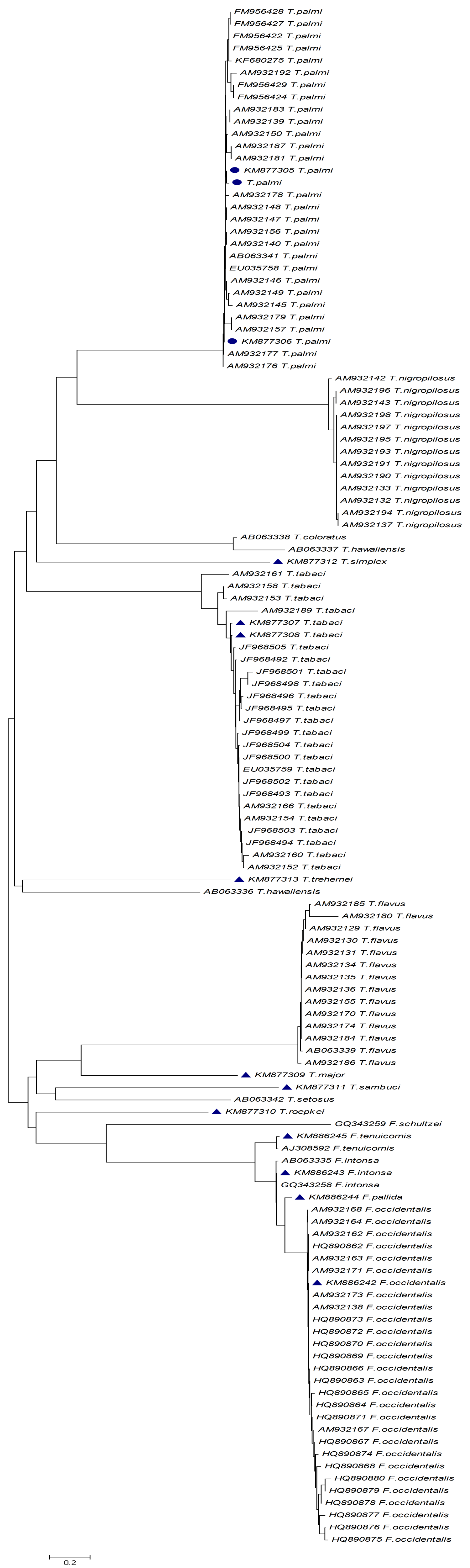

Supplement: S2 Fig — MEGA4 software [24] was used. Genetic distance is indicated. (TIF) [file pone.0122033.s002.tif]

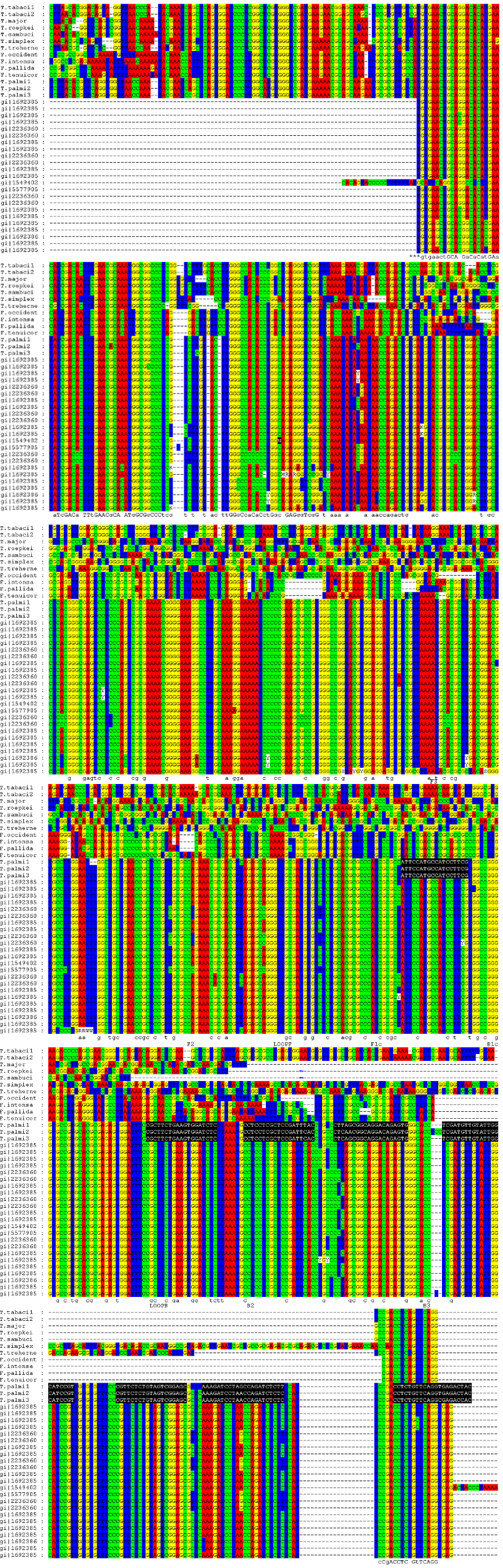

Supplement: S3 Fig — Loop-mediated isothermal amplification (LAMP) primer sequences are indicated (black background). The alignment was generated using the GeneDoc software [23]. (TIF) [file pone.0122033.s003.tif]
