# Supplementary material for: Detection of the Quarantine Species Thrips palmi by Loop-Mediated Isothermal Amplification
Source: PLoS One. 2015 Mar 20;10(3):e0122033. doi: 10.1371/journal.pone.0122033 (PMC4368663; doi:10.1371/journal.pone.0122033)
Supplement: S1 Table — (DOCX) [file pone.0122033.s004.docx]

**S1 Table. Sequences from the GenBank database used for designing PCR and LAMP primers**

| Species | Accession number |
| --- | --- |
| *T. palmi* | AM932141 |
| *T. palmi* | AB063341 |
| *T. palmi* | AM932146 |
| *T. palmi* | AM932148 |
| *T. palmi* | AM932147 |
| *T. palmi* | FM956425 |
| *T. palmi* | FM956422 |
| *T. palmi* | AM932177 |
| *T. palmi* | AM932176 |
| *T. palmi* | AM932149 |
| *T. palmi* | FM956428 |
| *T. palmi* | FM956427 |
| *T. palmi* | AM932156 |
| *T. palmi* | AM932140 |
| *T. palmi* | EU035758 |
| *T. palmi* | KF680275 |
| *T. palmi* | FM956429 |
| *T. palmi* | FM956424 |
| *T. palmi* | AM932178 |
| *T. palmi* | AM932150 |
| *T. palmi* | AM932183 |
| *T. palmi* | AM932139 |
| *T. palmi* | AM932188 |
| *T. palmi* | AM932187 |
| *T. palmi* | AM932181 |
| *T. palmi* | AM932145 |
| *T. palmi* | AM932179 |
| *T. palmi* | AM932192 |
| *T. palmi* | AM932157 |
| *T. palmi* | AM932182 |
| *T. palmi* | KF680274 |
| *T. tabaci* | EU035759 |
| *T. coloratus* | AB063338 |
| *T. havaniiensis* | AB063337 |
| *T. setosus* | AB063342 |
| *F. occidentalis* | GQ343256 |
| *F. occidentalis* | GQ343255 |
| *F. occidentalis* | GQ343254 |
| *F. intonsa* | GQ343258 |
| *F. schultzei* | GQ343259 |
| *Scirtothrips dorsalis* | GQ343260 |
| *Echinothrips americanus* | AJ303091 |
